# Acetazolamide-Induced Liver Injury: A Case Report and Literature Review

**DOI:** 10.7759/cureus.80536

**Published:** 2025-03-13

**Authors:** Aabid Mohiuddin, Fawaz Hussain, Ali Al-Ramadan, Kirthi K Lilley

**Affiliations:** 1 Department of Internal Medicine, Detroit Medical Center/Wayne State University, Detroit, USA; 2 Department of Gastroenterology, Wayne State University School of Medicine, Detroit, USA; 3 Department of Gastroenterology, Detroit Veterans Affairs Medical Center, Detroit, USA

**Keywords:** acetazolamide, carbonic anhydrase inhibitor, dili, drug-induced liver injury, hepatotoxicity

## Abstract

Acetazolamide is a carbonic anhydrase inhibitor, which has been very rarely associated with drug-induced liver injury (DILI). This report presents a case of an elderly male who developed severe but asymptomatic DILI associated with acetazolamide use. Severity was established based on markedly elevated serum transaminases. Subsequent rechallenge of acetazolamide at a higher dose resulted in increased severity of the elevations, further suggesting a causative effect. Liver function tests ultimately improved after acetazolamide discontinuation. This case underscores the need for early recognition and timely discontinuation of acetazolamide to prevent worsening liver injury and optimize clinical outcomes.

Only two prior documented cases of acetazolamide-induced DILI are found in the medical literature. A detailed review and comparison of those prior cases have been undertaken in the discussion of this case presentation.

## Introduction

Drug-induced liver injury (DILI) is a well-recognized adverse drug reaction characterized by hepatocellular, cholestatic, or mixed liver damage following medication exposure. Common culprits include antibiotics, nonsteroidal anti-inflammatory drugs (NSAIDs), and anticonvulsants. DILI diagnosis relies on the exclusion of other liver pathologies, with liver injury having occurred after drug exposure and improved upon discontinuation. Rechallenge with the offending drug is generally contraindicated due to the risk of rapid and severe recurrence of liver injury, which can lead to fulminant hepatic failure in some cases [1]. The following case highlights a rare example of DILI caused by acetazolamide, a carbonic anhydrase inhibitor with varying applications including as adjunctive diuretic therapy for generalized edema, adjunctive therapy for lowering intra-ocular pressure in glaucoma (by inhibiting aqueous humor production), and first-line therapy for reducing cerebrospinal fluid (CSF) production [2-4].

## Case presentation

A 66-year-old man with a history of stage IV lung adenocarcinoma with lumbar spinal metastases status post lumbar laminectomy two weeks prior was admitted to the medicine service for acute nausea and vomiting. His symptoms were attributed to an acute CSF leak identified during postoperative rehabilitation, as no other causes were identified. In addition to symptomatic care, he was placed on strict bedrest and started on a five-day course of acetazolamide 250 mg twice daily to reduce CSF production and mitigate the severity of the leak.

Three days post-treatment, a comprehensive metabolic panel revealed a significant elevation in liver enzymes consistent with a hepatocellular injury pattern. Alanine aminotransferase (ALT) increased from 12 U/L to 426 U/L (35-fold rise), aspartate aminotransferase (AST) from 16 U/L to 251 U/L (16-fold rise), and alkaline phosphatase (ALP) from 51 U/L to 278 U/L, while total bilirubin (T. Bili) remained stable at 0.34 mg/dL.

**Figure 1:**
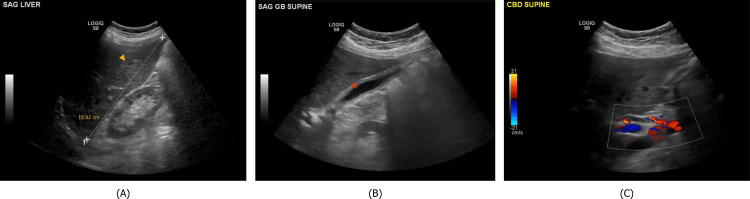
Liver ultrasonography obtained as part of investigation of the patient's elevated liver function tests. (A) Mild hepatomegaly is shown; the liver is measured at 18.92 cm. The yellow arrowhead points to a region of fatty metamorphosis. (B) Red arrowhead shows the gallbladder without any evidence of cholelithiasis. (C) Doppler imaging of the common bile duct reveals appropriate flow and no dilation.

The physical examination was unremarkable, with no signs of jaundice, hepatomegaly, or stigmata of chronic liver disease. Liver ultrasonography revealed mild hepatomegaly with hepatic steatosis, without cholelithiasis or ductal dilation (Figure 1). Viral hepatitis serologies were negative for acute or chronic infection of hepatitis A, B, and C, as well as Epstein-Barr virus (EBV), cytomegalovirus (CMV), and herpes simplex virus (HSV). Anti-nuclear, anti-mitochondrial, and anti-smooth muscle antibodies were negative. 

At this stage, no alternative etiology for the transient, asymptomatic liver injury was identified, increasing the suspicion of drug-induced hepatotoxicity. An initial medication review did not reveal any recent new-onset exposure to common hepatotoxic drugs, though nivolumab, an immune checkpoint inhibitor with potential liver toxicity, was noted in his history. The patient had last received an infusion one week prior to admission; however, no abnormality was noted on the follow-up laboratory evaluation. Furthermore, he had tolerated nivolumab infusions at regular intervals for the last five years without any previous hepatotoxicity. Therefore, it was deemed to be an unlikely causative agent.

Given that the patient was asymptomatic, and the initial workup was unremarkable, we adopted a conservative approach with serial laboratory monitoring and no acute intervention. The patient’s liver function tests (LFTs) slowly returned to normal over the next two weeks. A liver biopsy was not performed at this time due to the patient’s asymptomatic presentation, absence of progressive enzyme elevation, and expected improvement with conservative management.

The patient was soon discharged to a rehabilitation center for continued care; however, he was readmitted to our service after 10 days due to recurrent CSF leak. On readmission, LFTs were almost entirely within normal limits (ALT 66 U/L, AST 13 U/L, ALP 85 U/L, T. Bili 0.32 mg/dL), confirming complete resolution of prior liver injury. During his rehabilitative stay, acetazolamide was not administered, further supporting its role in the initial liver enzyme elevations. 

Strict bedrest protocols were reinitiated, and the neurosurgery team recommended restarting acetazolamide 250 mg thrice daily to manage intracranial pressure. On day 3, LFTs showed a dramatic rise, with ALT increasing nearly 100-fold from baseline, AST rising over 18-fold, and T. Bili showing a mild increase (ALT 1222 U/L, AST 293 U/L, ALP 216 U/L, T. Bili 0.82 mg/dL). As before, the patient was asymptomatic, and his physical examination was unremarkable. Liver biopsy showed inflammatory infiltrates in portal triads and lobules, with no evidence of autoimmune hepatitis or significant cholestasis, findings consistent with DILI.

Given the clear temporal relationship and exclusion of other etiologies, it was concluded that acetazolamide was the sole possible offending agent. After prompt discontinuation, LFTs declined and plateaued within 10 days before a further slow recovery toward the normal range, consistent with drug withdrawal. On outpatient follow-up after discharge, laboratory evaluation revealed further LFT recovery, with ALT and AST having returned to normal range. ALP remained slightly elevated, though this was suspected to reflect the progression of his metastatic bone lesions. A summary of the clinical course timeline with corresponding LFTs can be seen in Table 1. Several months after discharge, the patient enrolled in home hospice due to the progression of his primary malignancy.

**Table 1:** Timeline of patient's clinical course with correlated laboratory values. ALT, alanine aminotransferase; AST, aspartate aminotransferase; ALP, alkaline phosphatase; T. Bili, total bilirubin; CSF, cerebrospinal fluid; LFTs, liver function tests.

	ALT (reference range: 7-55 U/L)	AST (reference range: 8-48 U/L)	ALP (reference range: 40-129 U/L)	T. Bili (reference range: 0.1-1.2 mg/dL)
Outpatient | 2 days prior to Admission #1	12 U/L	16 U/L	51 U/L	0.30 mg/dL
	Admission #1 |Patient admitted to medicine service for management of post-lumbar laminectomy CSF leak			
Day 1 | Started on 5-day course of acetazolamide 250 mg twice daily	LFTs not obtained on admission			
Day 8 | 3 days post-completion of 5-day acetazolamide course	426 U/L	251 U/L	278 U/L	0.34 mg/dL
Day 15 | Discharged to in-patient rehabilitation center	103 U/L	16 U/L	87 U/L	0.24 mg/dL
	Admission #2 |Patient re-admitted to medicine service after 10 days at in-patient rehabilitation center due to recurrent CSF leak			
Day 1 | Started on 5-day course of acetazolamide 250 mg thrice daily	66 U/L	13 U/L	85 U/L	0.32 mg/dL
Day 3 | Acetazolamide discontinued due to suspicion of drug-induced liver injury	1222 U/L	293 U/L	216 U/L	0.82 mg/dL
	After discontinuation of acetazolamide, LFTs plateaued and began to slowly recover. The patient was ultimately discharged after clinical improvement			
Outpatient | 3 weeks after Admission #2	47 U/L	16 U/L	213 U/L	0.82 mg/dL

## Discussion

DILI presents with a wide spectrum of severity, ranging from asymptomatic mild transaminase elevations to acute liver failure [5]. Carbonic anhydrase inhibitors such as acetazolamide are very rare causes of clinically apparent DILI given the paucity of cases in the existing literature. The mechanism of hepatic injury due to acetazolamide is not well understood; however, it is proposed to be due to a hypersensitivity reaction from cross-reactivity to sulfonamide drug reactions given the similar molecular structure [6]. DILI remains a diagnosis of exclusion; therefore, other etiologies of hepatitis must be ruled out, including but not limited to acute viral hepatitis, autoimmune hepatitis, ischemic liver injury, acute Budd-Chiari syndrome, Wilson disease, and primary or secondary neoplasms [7]. 

In this patient, viral and autoimmune causes of liver injury were excluded, and imaging showed no biliary obstruction or hepatic masses. The only known hepatotoxic drug the patient was receiving was nivolumab. However, nivolumab-associated hepatotoxicity is most often seen at initial infusion, with a median onset of 39 days to adverse reaction [8]. Our patient had been receiving the infusions at regular intervals for more than five years, without any hepatotoxicity. It was therefore excluded as a likely cause, leaving acetazolamide as the drug of interest.

To assess causality, we applied the Naranjo Adverse Drug Reaction (ADR) Probability Scale, where a score greater than 9 indicates definite causality. This case scored 10 on the Naranjo ADR scale, as liver injury followed acetazolamide initiation, resolved after discontinuation, recurred upon re-administration, and worsened at a higher dose. In comparison, the Naranjo ADR score for the use of nivolumab in this patient was only 2, which correlates to only possible causality [9] (Table 2). To further assess acetazolamide causality, we also applied the revised electronic Roussel Uclaf Causality Assessment Method (RECAM), a validated tool for DILI assessment [10,11]. The RECAM score for this case was 12, suggesting a high likelihood of acetazolamide DILI. Thus, the totality of evidence strongly supports acetazolamide as the most likely cause of DILI in this patient's care. 

**Table 2:** Comparison of Naranjo Adverse Drug Reaction Probability Scale scores for nivolumab and acetazolamide use in this patient case. Ref. [9].

	Nivolumab	Acetazolamide
1. Are there previous conclusive reports on this reaction? (Yes=1|No=0|Unsure=0)	1	1
2. Did the adverse event appear after the suspected drug was administered? (Yes=2|No=-1|Unsure=0)	0	2
3. Did the adverse event improve when the drug was discontinued or a specific antagonist was administered? (Yes=1|No=0|Unsure=0)	0	1
4. Did the adverse event reappear when the drug was re-administered?(Yes=2|No=-1|Unsure=0)	-1	2
5. Are there alternative causes (non-medication) that could on their own have caused the reaction?(Yes=-1|No=2|Unsure=0)	2	2
6. Did the reaction reappear when a placebo was given? (Yes=-1|No=1|Unsure=0)	0	0
7. Was the drug detected in blood or other fluids in concentrations known to be toxic?(Yes=1|No=0|Unsure=0)	0	0
8. Was the reaction more severe when the dose was increased or less severe when the dose was decreased? (Yes=1|No=0|Unsure=0)	0	1
9. Did the patient have a similar reaction to the same or similar drugs in any previous exposure? (Yes=1|No=0|Unsure=0)	0	0
10. Was the adverse event confirmed by any objective evidence? (Yes=1|No=0|Unsure=0)	0	1
Total	2	10

A potential weakness in the case was noted in the absence of LFTs being obtained on the first day of his initial admission (as indicated in Table 1). However, given the short interval between outpatient LFTs and admission (two days) and the lack of intervention or novel drug administration during that period, any impact on the case findings is likely minimal.

Only two prior case reports of acetazolamide-induced DILI exist in the literature. In 1967, a 54-year-old British man with no prior medical history presented with failing vision attributed to glaucoma and was prescribed oral acetazolamide 500 mg daily. He returned one month later with abdominal pain, marked jaundice, extreme right upper-quadrant abdominal tenderness, and progressive encephalopathy. Laboratory evaluation revealed cholestatic liver injury (ALT 180 U/L, ALP 44 U/L, T. Bili 10.9 mg/dL). He ultimately failed to respond to supportive treatment and expired with cholestatic jaundice listed as the cause of death. Acetazolamide was the sole drug he had received [12]. A 2013 report highlighted the case of a 44-year-old Turkish woman who was prescribed acetazolamide 250 mg daily for the management of pseudotumor cerebri. After two weeks, she developed nausea and jaundice, with laboratory evaluation revealing cholestatic liver injury [ALT 492 U/L, AST 735 U/L, ALP 44 U/L, T. Bili 8.93 mg/dL (direct 8.91 mg/dL)]. Laboratory testing for viral hepatitis, autoimmune hepatitis, Wilson’s disease, and hemochromatosis were all negative, and abdominal imaging revealed only benign findings. After cessation of acetazolamide, her symptoms and markers of liver injury recovered completely after three weeks [13]. 

Unlike prior cases, this patient remained asymptomatic and did not develop jaundice. A peak R ratio of 17.1, calculated as [(ALT/Upper Limit Normal (ULN)) ÷ (ALP/ULN)], confirmed a hepatocellular injury pattern, distinguishing this case from previous cholestatic presentations. Table 3 summarizes the key clinical features and biochemical profiles of all three reported cases of acetazolamide-induced DILI.

**Table 3:** Summary of reported cases of acetazolamide-induced DILI, comparing injury patterns, dosing, and clinical course. DILI, drug-induced liver injury.

Case report	Predominant pattern of liver injury	Dose administered	Duration of exposure	Clinical presentation
Kristinsson (1967) [12]	Cholestatic	500 mg daily	30 days	Abdominal pain, marked jaundice, encephalopathy. Fatal outcome due to cholestatic liver failure.
Başar et al. (2013) [13]	Cholestatic	250 mg daily	14 days	Nausea, jaundice
Mohiuddin et al. (2025) (this study)	Hepatocellular	Initial course: 250 mg twice daily; subsequent course: 250 mg thrice daily	Initial 5-day course; subsequent 5-day course (aborted on day 3)	Asymptomatic

## Conclusions

This case presents a rare instance of severe acetazolamide-induced DILI with a predominantly hepatocellular pattern, distinguishing it from previously reported cholestatic cases. Clinicians should maintain a high index of suspicion for acetazolamide-induced liver toxicity, particularly in patients with unexplained transaminase elevations. LFTs should be measured prior to initiation of acetazolamide and routinely monitored thereafter. Early detection, immediate discontinuation, and close monitoring are essential to prevent progression to severe liver injury and optimize clinical outcomes. Further research is warranted to elucidate the underlying mechanisms of acetazolamide-induced hepatotoxicity and identify individual susceptibility factors.
